# Personal Mention

**Published:** 1919-05

**Authors:** 


### PERSONAL MENTION.

Dr. Frank Litten, of Austin, was found dead on March 16th, near Austin, the coroner's finding being that he died from self-administered poison. Dr. Litten was one of Austin's best known physicians. He overworked during the influenza epidemic and had been suffering from a physical and nervous breakdown due to the long strain upon him. His untimely death is a matter of general regret.

Dr. Chambers, of Liberty, Texas, has been selected as one of the two medical officers from his division to attend the University of Lyons, France.

Dr. Ethel T. Heard, of Galveston, has returned from France, bringing with her a war baby and a war puppy as souvenirs of her nineteen months war work in France.

Dr. O. J. Cooke, a well known surgeon and physician of San Antonio, who has just received his service discharge, will go to Chili as physician-surgeon for a big copper company.

Dr. A. M. Meal, of Celeste, Texas, was severely injured recently when a Katy train ran into his car.

Dr. Chas. M. McMillan, formerly of Plantersville, having received his discharge is now located in Waco, and has offices in the Praetorian building.

Dr. J. S. Wilkins, of Hot Springs, Ark., who has been a captain in the service in France, has returned to Hot Springs and resumed the practice of medicine with his former partner, Dr. Breedlove.

Dr. J. Spencer Davis, having been released from military service, has returned to his home in Dallas where he has resumed his practice, making diseases of children a specialty.

Dr. James Sanders, for forty years a physician at Orange, and said to have been the oldest Freemason in Texas, died recently at Beaumont, where he had been a few months.

Dr. T. A. King, of Vernon, has been appointed a member of the State Board of Medical examiners of Texas, in place of Dr. Berry of San Antonio.

Dr. L. L. Lacey, of Austin, aged 68, died recently after a short illness. He was one of Austin's prominent physicians.

Dr. Joe Wolfe, who has been serving as a captain in France and Germany, will re-enter the practice at his old home in Van Alstyne, Texas.

Dr. Wallace Ralston, who was a major in the army, has returned to Houston after a year's absence to resume his practice as an eye specialist. Prior to his discharge January 1, Major Ralston was in command of the base hospital at Camp Jackson, S. C., for three months.

Under the heading "San Antonio Woman Saves One Thousand Lives," the San Antonio Evening News recently carried a large portrait of Dr. Clara G. Cook, formerly of Hearne, Texas, and an article covering several columns of that paper regarding work of Dr. Cook in France.

Two of Beeville's physicians who entered the Officers' Medical Reserve Corps have returned to resume their practice. Capt. Houston Neely was the first to return, and he was followed by Lieut. A. J. Turner.

Doctor J. E. Langston, late of the government service, and formerly of Cisco, is now located in De Leon, having opened offices there in the Sellers Drug Store.

Dr. Carl T. Steen has returned from training in Camp Pike, Little Rock, Arkansas, and is now located in Wichita Falls.

The board of trustees of the State School for the Deaf elected Dr. F. B. Shufford of Wolfe City, Hunt County, superintendent of the institution and he will qualify and enter upon the discharge of his duties March 1.

Dr. Beverley M. Young, appointed under former Governor Ferguson as superintendent of the southwestern asylum for the insane, was re-elected for another term at the meeting of the board of managers February 6.

Doctor John Dee Jackson, formerly of the Research Hospital of Kansas City, has recently moved to Kerrville. Dr. Jackson has been on duty at the laboratories of Yale University and goes to Kerrville to associate with Dr. Secor as a diagnostic specialist

Dr. and Mrs. A. J. Turner of Beeville, Texas are now located at Norfolk, Virginia, where Lieut. Turner was sent shortly after being accepted by the Reserve Corps.

Dr. Joe S. Wooten of Austin, Texas, who has been critically ill for some time, due to a breakdown caused from overwork, is very much improved, much to the delight of his many friends over the state.

Dr. and Mrs. A. M. Kotzebue of Moulton have moved to San Antonio to reside.

---

WRONG DIAGNOSIS:—Doctor—Get my kit, quick. Some fellow telephones in a dying voice that he can't live without me.

His Wife—Just a moment. That call is for daughter, dear.

